# What Is the Diagnostic Accuracy of the Duck Walk Test in Detecting Meniscal Tears?

**DOI:** 10.1007/s11999-017-5475-6

**Published:** 2017-08-14

**Authors:** A. Van der Post, J. C. A. Noorduyn, V. A. B. Scholtes, E. L. A. R. Mutsaerts

**Affiliations:** 1 Universiteit van Amsterdam, Meibergdreef 9, 1105 AZ Amsterdam, The Netherlands; 2 Onze Lieve Vrouwe Gasthuis, Amsterdam, The Netherlands

## Abstract

**Background:**

Clinical weightbearing provocation tests, like the duck walk test, may be of value in diagnosing or screening for medial meniscal tears. However, evidence of the diagnostic accuracy of the duck walk test is lacking.

**Questions/purposes:**

(1) To determine the sensitivity and specificity of the duck walk test in diagnosing medial meniscal tears. (2) To determine whether tear location, tear cause (traumatic versus degenerative), and ACL insufficiency were associated with differences in the sensitivity and specificity of the test.

**Methods:**

A convenience sample of 136 patients of all ages was retrospectively analyzed by evaluating the outpatient knee clinic appointment list of one orthopaedic surgeon for patients with a broad range of knee injuries who had a prior MRI before (24%) or after (76%) physical examination and had a duck walk test stated in their patient records. Of 230 patients with MRI requested by one orthopaedic surgeon attributable to knee complaints, 136 (59%) fulfilled the inclusion criteria; 70 (52%) patients were male and 66 (49%) were female, with a mean age of 42 (± SD 14) years. The duck walk test was performed in case of suspected meniscal injury, based on mechanism of injury, general joint line pain, and/or mechanical complaints (ie, locking, giving away). The test is performed by squatting and “waddling” before rising and is positive in case of general joint line pain or painful “clicking”. Interobserver repeatability was not evaluated, but the test is well defined and leaves little room for difference in interpretation. Diagnostic accuracy measures were evaluated. Since the convenience sample in this study consisted of patients who had a duck walk test and MRI, and a positive result of the duck walk test almost certainly increased the probability that MRI would be ordered in the majority (76%) of the patients, the test properties calculated here—especially sensitivity—should be considered inflated.

**Results:**

The calculated sensitivity of the duck walk test was 71% (95% CI, 59%–81%) and there was low specificity of 39% (95% CI, 27%–52%). We found no difference in sensitivity between medial (67%; 95% CI, 51%–80%) and lateral (76%; 95% CI, 50%–92%; p = 0.492) meniscal tears. With the numbers available, we compared these patients with patients without a history of trauma and with an intact ACL. We found no difference among patients with traumatic tears (79%; 95% CI, 59%–91%; p = 0.253) and in patients with ACL tears (77%; 95% CI, 46%–94%; p = 0.742).

**Conclusions:**

Because of the issue of verification bias, the actual sensitivity of this test in practice is likely much lower than the calculated sensitivity we observed. In addition, the test did not seem to perform better in patients with trauma or ACL insufficiency, nor was it more effective in detecting medial than lateral tears, although the numbers on some of those comparisons were rather small. Based on these results, we conclude that used alone, the duck walk test likely has little value in practice as a screening test. However, it is conceivable that it could be used in combination with other provocative tests for screening purposes. Future studies might consider using it as a means to best identify which patients should undergo MRI for the possibility of a meniscal tear.

**Level of Evidence:**

Level III, diagnostic study.

## Introduction

Meniscal tears are a common cause of functional impairment of the knee with an incidence of approximately two per 1000 patients per year in The Netherlands [7]. History-taking and physical examination are the initial steps in diagnosing meniscal tears. The physical examination includes nonweightbearing and weightbearing meniscal provocation tests. The most commonly used traditional nonweightbearing tests are the McMurray’s test, joint-line tenderness, and the Apley grind test [2, 10, 14, 16, 19]. A frequently used weightbearing test is the Thessaly test and high sensitivity and specificity have been reported [2, 10, 11]. If surgery is contemplated, the diagnosis often is confirmed with MRI. However, routine or common use of MRI as the initial step in the evaluation of the painful knee may contribute to rising healthcare costs and should be limited to specific indications [15, 22, 23].

In 1957, Harold M. Childress described a new weightbearing test for diagnosing posterior lesions of the medial meniscus based on his clinical experiences [4]. The majority of his patients with injury to the posterior horn of the medial meniscus reported a loud clicking in the knee associated with sharp pain at the posteromedial portion of the joint when squatting down resulting from impingement of the meniscus at full flexion. Based on these observations, he formulated a meniscal provocation test, the “duck waddle” (now commonly termed the duck walk) test. The patient squats and “waddles” from side to side and back and forth before rising. A positive sign is evidenced by incomplete flexion limited by pain at the posteromedial joint line or by “clicking” in this region associated with discomfort. However, joint line pain in general is a common clinically used end point for a positive test result [8, 16, 17]. Although the duck walk test may be clinically useful for differentiating knee injuries, evidence is lacking regarding the diagnostic accuracy of this test. Although the duck walk test for meniscal injuries (or its synonyms, Childress’ sign or squat test) has been described in several studies [8, 16, 18, 20], we were able to find only one diagnostic study evaluating the duck walk test in ACL-insufficient knees [17]. In that study, Pookarnjanamorakot et al. [17] compared Apley’s test, Childress’ sign (squat test), McMurray’s test, Steinmann I sign, joint line tenderness, and Merke’s sign. The duck walk test had the highest sensitivity (68%) for pinpointing meniscal tears in patients with ACL injuries.

In the United States, the diagnostic MR imaging rate has nearly tripled during a 10-year period and is associated with rising healthcare costs [22]. Because MRI is expensive and access is limited in some healthcare systems, diagnostic tests like the duck walk test can be helpful to evaluate the probability of meniscal injury and thus be used in decision-making regarding MRI use. The test does not require difficult technical skills and has a plausible theory of detecting even small posterior tears by virtue of wringing the posterior horn at full flexion.

Intrigued by Childress’ theory behind the duck walk test, but hampered by the lack of diagnostic studies, we sought to determine the sensitivity and specificity of the duck walk test for diagnosing a meniscal tear. Secondarily, we examined whether the result of the duck walk test was influenced by the location of the tear (medial or lateral), the cause of the lesion (traumatic versus degenerative), and the presence or absence of ACL insufficiency.

## Patients and Methods

Our study complies with the Standards for Reporting Diagnostic Accuracy guidelines designed to improve the completeness and transparency of reporting of diagnostic accuracy studies [3].

This study was approved by the local medical ethical committee (WO 15.024, March 2, 2015).

This single-center retrospective cohort study comprised a convenience series of patients who were evaluated at the outpatient knee clinic of the Onze Lieve Vrouwe Gasthuis (OLVG) in Amsterdam between January 2013 and December 2014. The appointment lists of one orthopaedic surgeon (ELARM) were searched for patients who had a prior MRI (reference standard) and whose records documented the results of the duck walk test (index test). Bearing this diagnostic study in mind, the test results were separately stated in patients’ records as often and as accurately as possible during this time. This clinic sees a general population of patients of all ages with a broad range of knee complaints, ranging from acute sports injury to degenerative changes. The duck walk test was performed in case of suspected meniscal injury, based on mechanism of injury, general joint line pain, and/or mechanical complaints (ie, locking, giving way). Inclusion criteria for the study were men and women of all ages who presented with knee pain, where the result of the duck walk test was clearly stated in the medical record, and MRI was available as a result of symptoms related to an intraarticular knee disorder. In 103 (76%) patients, MRI was ordered by the orthopaedic surgeon (EM) after the physical examination, and in 33 instances (24%) patients were referred by an outside provider, already having had MRI. In these cases, the MRI results were known before physical examination. Exclusion criteria included (1) a period between MRI and clinical physical examination exceeding 3 months; (2) a physical examination performed in the acute or subacute phase of the injury (< 6 weeks, to avoid false positive outcomes); or (3) if the duck walk test was stated untestable (owing to joint effusion [n = 1] or patient’s inability to squat [n = 1]). All data were extracted from the patients’ electronic medical records. During the time in question, 1135 patients had MRI owing to knee complaints, of which 230 were requested by the orthopaedic surgeon (EM) and therefore were evaluated in this study. After evaluation, 136 (136 of 230; 59%) patients fulfilled the criteria to be included in the study. Of the 136 patients, 70 (52%) were male and 66 (49%) were female, with a mean age of 42 (± SD 14) years (Table 1).

**Table 1:** Patient characteristics

Variable	All patients (n = 136)	p Value
Age (years), mean (± SD)	42 (± 14)	NA
Sex, number (%)		
Male/female	70 (52)/66 (49)	0.732
Affected knee, number (%)		
Left/right	72 (53)/64 (47)	0.493
Trauma, number (%)		
Yes/no	57 (42)/79 (58)	0.059
Locking, number (%)		
Yes/no	28 (24)/87 (76)^*^	NS
Previous knee surgery, number (%)		
Yes/no	33 (37)/57 (63)^†^	0.011

*Twenty-one missing; ^†^46 missing; NA = not applicable; NS = nonsignificant.

The duck walk test was administered by one experienced orthopaedic surgeon (ELARM) in all patients and was performed as originally described [4] with a minor variation. Owing to inconsistent documentation of the clinical results in medical records, a distinction of test results was made, dividing them into a “squatting” and “squatting and duck-walking” component. Both components were scored separately in our data extraction as either “positive” or “negative,” after which an overall duck walk test score was used for the data analysis. The duck walk test was interpreted as positive if the clinical file reported that the patient could not perform, was limited, or experienced pain in performing the “duck walk test,” the “squatting,” or the “squatting and duck-walking” component. A negative duck walk test was scored if the clinical file reported that the patient was able to perform the “duck walk test” or the “squatting and duck-walking” component without distinctive pain. The duck walk test results were graded as incomplete if the clinical file solely reported that the patient could perform the “squatting” component and therefore was not used for further analyses. In the current study, reproducibility of the test was not evaluated because of the retrospective study design. However, the test is well defined and we believe it leaves little room for difference in interpretation of test results. It requires less technical skill from clinicians than many other provocative meniscal tests and patients are familiar with the movement. However, misinterpretation is possible if the clinician does not recognize poor execution attributable to for diminished mobility, strength, or joint effusion, for example.

It is likely that patients with suspected knee injury based on history and physical examination undergoing the duck walk test were more likely to have MRI, therefore increasing the possibility of verification bias. Because of this, the test properties calculated here should be considered inflated, with an overestimation of sensitivity and an underestimation of specificity [5, 9, 12, 24, 25]. In practice, the duck walk test would be used as a screening test and therefore sensitivity is the most important diagnostic value, in comparison to MRI, which is used as the confirmatory test and where specificity is most important.

The MRI was considered the reference standard for diagnosing the meniscal tear. All MR images were routinely assessed by the attending radiologist with concise clinical information available, but unaware of index test results. If presence, location, or type of tear remained unclear after studying the radiologist’s assessment, a second assessment was obtained with the assessor (EM) blinded for index test results. Meniscal tears were classified as present when observed on the MR images and defined as an increased signal intensity unequivocally contacting the joint surface in two or more images. Thereafter, the tear pattern was classified using the International Society of Arthroscopy, Knee Surgery and Orthopaedic Sports Medicine (ISAKOS) classification [1]. Furthermore, an option, “degenerative,” was added to the ISAKOS classification system for the degenerative tear pattern. Overall, 69 (51%) patients were diagnosed with one or more meniscal tears. The majority of meniscal tears were medial and the most common types of tears were degenerative (23%), complex (22%), and horizontal (20.0%) tears (Table 2). Patients with multiple tears were counted as one and were excluded in subgroup analyses. Furthermore, the MRI findings showed that 116 (85%) patients were diagnosed with intraarticular knee disorders consisting of a meniscal tear and/or diverse intraarticular disorders (ranging from cysts and loose bodies to chondropathy and bone marrow edema), leaving 20 (15%) patients with no disorder described in their patient records. Joint effusion was present in 31 (24%, data missing for six patients) patients and ACL injury was present in only 26 (19%) patients.

**Table 2:** Types of meniscal tears identified on MRI

Type of meniscal tear	All tears (%) (n = 69)	Medial tears (%) (n = 43)	Lateral tears (%) (n = 17)
Longitudinal	3 (4)	1 (2)	2 (12)
Bucket handle	4 (6)	3 (7)	1 (6)
Horizontal	14 (20)	13 (30)	1 (6)
Flap	1 (1)	1 (2)	0 (0)
Radial	7 (10)	3 (7)	4 (24)
Degenerative	16 (23	11 (26)	5 (29)
Complex	15 (22)	11 (26)	4 (24)
Multiple (mediolateral)	9 (13)	NA	NA
Total	69 (100)	43 (62)	17 (25)

NA = not applicable.

### Statistical Analysis

All statistical analyses were performed using SPSS Statistics Version 22.0.0.1 (IBM Corp, Armonk, NY, USA). Descriptive statistics were used to describe all patient demographics, means, and SDs for continuous normally distributed data, and median and interquartile range for continuous nonnormally distributed data. Frequencies and percentages were used for categorical data. Significant differences were tested with the unpaired t-test for normally distributed data, the Mann-Whitney U test for nonnormally distributed data, and the chi square test for ordinal data. When the chi square test showed expected counts of less than five in any of the cells, Fisher’s exact test was used instead. A probability less than 0.05 was considered significant.

The results of the duck walk test were plotted against the results of the MRI as the reference standard. Diagnostic accuracy measures were calculated from crosstables available at www.vassarstats.net. Additionally, all patients were divided in subgroups based on the location of the tear (anterior [n = 7] versus posterior [n = 48] horn of the medial meniscus and medial [n = 43] versus lateral [n = 17] meniscus), onset of knee pain (traumatic [n = 57] versus degenerative [n = 79]) and ACL injury (ACL tear [n = 26] versus no ACL tear [n = 110]). Diagnostic accuracy measures for each subgroup were determined and differences were presented by using descriptive statistics. When analyzing a subgroup, other tears were excluded and patients with no meniscal tears (n = 67) were used as a negative test result. In each subgroup, significant differences in sensitivity and specificity were calculated.

## Results

The calculated sensitivity of the duck walk test was 71% (95% CI, 59%–81%) and the specificity was 39% (95% CI, 27%–52%). This results in a positive predictive value of 54% (95% CI, 44%–65%) and a negative predictive value of 57% (95% CI, 41%–71%) (Table 3). As mentioned earlier, this calculated sensitivity is likely much higher than actual sensitivity because of the issue of verification bias (the probability that a positive test increased the likelihood that an MRI would be ordered) [5, 9, 12, 24, 25].

**Table 3:** Two-by-two crosstable results of the duck walk test for meniscal lesions and corresponding diagnostic values

Crosstable results		Meniscal tear on MRI		
		Yes	No	Total
Duck walk test				
Positive		49	41	90
Negative		20	26	46
Total		69	67	136
Prevalence (%)	51 (42–59)			
Sensitivity (%)	71 (59–81)			
Specificity (%)	39 (27–52)			
PPV (%)	54 (44–65)			
NPV (%)	57 (41–71)			
+LR	1.16 (0.91–1.48)			
−LR	0.75 (0.49–1.13)			
DOR	1.55 (0.76–3.18)			

Values in parentheses are 95% CI; PPV = positive predictive value; NPV = negative predictive value; +LR positive likelihood ratio; −LR = negative likelihood ratio; DOR = diagnostic odds ratio.

With the numbers available, tear location (anterior versus posterior horn of the medial meniscus, and medial versus lateral meniscus), tear etiology (traumatic versus degenerative), and status of the ACL were not associated with differences in the performance of the duck walk test. The sensitivity of the duck walk test for detecting medial meniscal tears in either the posterior horn (67%; 95% CI, 51%–79%) or the anterior horn (71%; 95% CI, 30%–95%; p = 1.000) was not different with the numbers available. Likewise, the test’s sensitivity did not differ with the numbers available between lateral meniscus tears (76%, 95% CI, 50%–92%) and medial meniscus tears (76%, 95% CI, 51%–80%; p = 0.492) (Table 4). The sensitivity of 79% (95% CI, 59%–91%) in patients with traumatic tears was no different, with the numbers available, from the 66% (95% CI, 49%–79%; p = 0.253) observed in patients with degenerative tears (Table 5). Finally, the sensitivity of 77% (95% CI, 46%–94%) in patients with ACL tears was no different, with the numbers available, from the 70% (95% CI, 56%–81%; p = 0.742) observed in patients without ACL tears. The specificity was less than 50% for all subgroups and no differences were observed among them in terms of specificity.

**Table 4:** Diagnostic values of the duck walk test in detecting meniscal tears in different locations

Diagnostic values	Posterior horn (n = 48)	Anterior horn (n = 7)	Medial meniscus (n = 43)	Lateral meniscus (n = 17)
Prevalence (%)	42 (33–51)	9.5 (4.2–19)	39 (30–49)	20 (13–31)
Sensitivity (%)	67 (51–79)	71 (30–95)	67 (51–80)	76 (50–92)
Specificity (%)	39 (27–52)	39 (27–52)	39 (27–52)	39 (27–52)
PPV (%)	44 (32–56)	11 (4.1–24)	41 (30–54)	24 (14–38)
NPV (%)	62 (46–76)	93 (75–99)	65 (48–79)	87 (68–96)
+LR	1.09 (0.83–1.44)	1.17 (0.70–1.94)	1.10 (0.83–1.46)	1.25 (0.90–1.73)
−LR	0.86 (1.55–1.34)	0.74 (0.22–2.49)	0.84 (0.52–1.35)	0.61 (0.25–1.50)
DOR	1.27 (0.58–2.75)	1.59 (0.29–8.78)	1.31 (0.59–2.94)	2.06 (0.61–7.01)

Values in parentheses are 95% CI; PPV = positive predicting value; NPV = negative predicting value; +LR = positive likelihood ratio; −LR = negative likelihood ratio; DOR = diagnostic odds ratio.

**Table 5:** Diagnostic values of the duck walk test in detecting traumatic or degenerative meniscal tears and with or without ACL tear

Diagnostic values	Traumatic (n = 57)	Degenerative (n = 79)	With ACL tear (n = 26)	Without ACL tear (n = 110)
Prevalence (%)	49 (36–63)	52 (40–63)	50 (30–70)	51 (41–60)
Sensitivity (%)	79 (59–91)	66 (49–79)	77 (46–94)	70 (56–81)
Specificity (%)	45 (27–64)	34 (20–51)	46 (20–74)	37 (25–51)
PPV (%)	58 (41–73)	52 (38–66)	59 (33–81)	53 (41–65)
NPV (%)	68 (43–86)	48 (29–68)	67 (31–91)	54 (37–70)
+LR	1.42 (0.97–2.08)	1.00 (0.73–1.38)	1.43 (0.80–2.56)	1.11 (0.85–1.45)
−LR	0.48 (0.22–1.06)	1.00 (0.59–1.69)	1.50 (0.16–1.61)	0.82 (0.52–1.30)
DOR	2.98 (0.93–9.52)	1.00 (0.40–2.54)	2.86 (0.53–15.47)	1.35 (0.61–2.98)

Values in parentheses are 95% CI; PPV = positive predicting value; NPV = negative predicting value; +LR = positive likelihood ratio; −LR = negative likelihood ratio; DOR = diagnostic odds ratio.

## Discussion

Meniscal tears are a common cause of functional impairment of the knee. Because MRI is expensive, diagnostic physical tests can be a helpful screening tool in decision-making regarding MRI use. In practice, the duck walk test would be used as a screening test and therefore sensitivity is the most important diagnostic value. Intrigued by Childress’ theory behind the duck walk test we sought to determine its diagnostic value. In addition, we examined whether the results were influenced by location of the tear (medial or lateral), cause of the lesion (traumatic versus degenerative), and the presence or absence of ACL insufficiency. The results of this study showed a calculated sensitivity of 71% (95% CI, 42%–59%). However, because of verification bias, actual sensitivity would be considered too low to use this test in practice. Calculated specificity was 39% (95% CI, 27%–52%), but this probably is less relevant since it concerns a screening test. No differences were found when comparing between tear locations, tear etiology, and presence of an ACL tear.

We are aware of the limitations of this study, with a retrospective study design being perhaps the primary limitation. The major bias this introduced was verification bias. It is certain that in some—perhaps many—patients studied here, a positive duck walk test would have made it more likely that confirmatory MRI would be ordered. This would be expected to cause the calculated sensitivity we report here to overestimate—perhaps by a considerable margin—the actual sensitivity of the test in practice [5, 9, 12, 24, 25]. While this bias also might cause us to underestimate the specificity of the test, this matters little, since the duck walk test is a screening test, not a confirmatory test (in practice, MRI is the preferred confirmatory test). In addition, because of the retrospective study design, some patients could not be included because of missing data; it is difficult to assess the effect of that on our study’s results, but there is little reason to conclude that this problem would result in the test performance being better in practice than what we observed here. Furthermore, this study is affected by assessor bias. In 33 (24%) patients the orthopaedic surgeon was already aware of the MRI results when performing the duck walk test. Interpretation of the duck walk test therefore could be biased and also could have led to overestimation of the test’s accuracy [13].

This study used the MRI findings as the reference standard instead of arthroscopy. According to a systematic review by Crawford et al. [6], MRI has a sensitivity of 82.5% and a specificity of 92.8% compared with arthroscopy. Although MRI is generally accepted as the reference standard in clinical research on meniscal tears, this may have led to deflated specificity because some tears could have been missed on the MR images. Again, this is less important when considering this examination for use as a screening test. In addition, the number of patients with present meniscal tears in all subgroups is relatively low, creating large confidence intervals and making it less likely to detect a difference between the subpopulations (eg, tear location, tear etiology, and status of the ACL).

Childress originally described the duck waddle test in medial meniscal tears, and described it as positive if incomplete flexion was limited by pain at the posteromedial joint line or by “clicking” in this region associated with discomfort [4]. In current clinical use however, joint line pain in general is the more commonly used end point [8, 16, 17]. We also used general joint line pain as an end point in this study, which differs from Childress’ original description. This also might have inflated sensitivity, although we do not expect this to be a major influence since we included lateral meniscal tears as well. In contrast to Childress’ hypotheses of higher diagnostic accuracy measures in medial meniscal tears, we found no difference. In this cohort no painful clicking was recorded, but because of the retrospective study design we cannot conclude no clicking was present.

The calculated sensitivity of 71% for the duck walk test (which, as noted, likely substantially overestimates the test’s sensitivity in practice) and its low specificity of 39% makes this test unlikely to be helpful in practice, at least when used in isolation. To our knowledge, this is the first extensive study of the diagnostic value of the duck walk test. The most recent systematic review on diagnostic values for meniscal provocation tests by Smith et al. [21] included a meta-analysis on several specific tests. These diagnostic accuracy measures are suitable for comparison since, as Smith et al. [21] indicate, verification bias was the greatest source of bias in all but one study and thus, the calculated sensitivities are probably an overestimation as well. The Thessaly test in 20° also is described as a weightbearing meniscal provocation test and perhaps therefore most comparable to the duck walk test from the current study. Smith et al. [21] calculated a pooled sensitivity for the Thessaly 20° test of 75% (95% CI, 53%–89%), which is similar to the duck walk test’s calculated sensitivity of 71% (95% CI, 59%–81%). McMurray’s test and joint line tenderness are the most frequently used meniscal tests and pooled sensitivity of 61% (95% CI, 45%–74%) and 83% (95% CI, 73%–90%) respectively were calculated, which indicates relatively lower and higher sensitivity compared with the duck walk test. Therefore, future studies might consider evaluating diagnostic accuracy measures when used in combination with other provocative tests for screening purposes in a population of patients observed prospectively who will undergo MRI regardless of the results of these tests, to minimize the risk of verification bias. The duck walk test’s specificity at 39% (95% CI, 27%–52%) seems lower than those of the Thessaly 20° test (87%; 95% CI, 65%–96%), McMurray’s (84%; 95% CI, 69%–92%), and joint line tenderness (83%; 95% CI, 61%–94%). However, specificity is less relevant, as both tests are used in practice as screening tests, not confirmatory tests.

We did not find that the duck walk test performed better in any particular subpopulation of patients. With the numbers available, it was not more sensitive in detecting medial versus lateral tears (or anteromedial versus posteromedial tears), or tears in patients with differing diagnoses. Comparing our results with those of other studies is challenging because specific diagnostic studies or accuracy values were only mentioned in the study by Pookarnjanamorakot et al. [17], a cross-sectional prospective study of 100 patients with an ACL injury and symptoms of instability. They compared several meniscal tests and although it was not a diagnostic study on the duck walk test specifically and tests are solely described for patients with ACL injury, it is the only study suitable for comparison with results of our study. Because substantial differences were found in the prevalence (50% versus 75%) between the two studies, predictive values could not be compared.

Because of the issue of verification bias, the actual sensitivity of this test in practice is likely much lower than the calculated sensitivity we observed of 71%; in addition, the test did not seem to perform better in patients with trauma or ACL insufficiency, and it was no more effective in detecting medial than lateral tears, although the numbers for some of those comparisons were rather small. Based on these results, we conclude that used alone, the duck walk test probably has little value in practice as a screening test. However, it is conceivable that it could be used in combination with other provocative tests for screening purposes; future studies might consider using it as a means to best identify which patients should undergo MRI for the possibility of meniscal tear.
